# GAPDH suppresses adenovirus-induced oxidative stress and enables a superfast production of recombinant adenovirus

**DOI:** 10.1016/j.gendis.2024.101344

**Published:** 2024-05-31

**Authors:** Guozhi Zhao, Piao Zhao, Yonghui Wang, Hui Zhang, Yi Zhu, Jiamin Zhong, Wulin You, Guowei Shen, Changqi Luo, Ou Mei, Xingye Wu, Jingjing Li, Yi Shu, Hongwei Wang, William Wagstaff, Hue H. Luu, Yang Bi, Lewis L. Shi, Russell R. Reid, Tong-Chuan He, Li Jiang, Wei Tang, Jiaming Fan, Ziwei Tang

**Affiliations:** a Departments of Urology, Endocrinology, Orthopedic Surgery, and Gastroenterological Surgery, The First Affiliated Hospital of Chongqing Medical University, Chongqing 400016, China; b Molecular Oncology Laboratory, Department of Orthopaedic Surgery and Rehabilitation Medicine, The University of Chicago Medical Center, Chicago, IL 60637, USA; c Ministry of Education Key Laboratory of Diagnostic Medicine, and Department of Clinical Biochemistry, School of Laboratory Medicine, Chongqing Medical University, Chongqing 400016, China; d Department of Geriatrics, Xinhua Hospital, Shanghai Jiao-Tong University School of Medicine, Shanghai 200000, China; e The Breast Cancer Center, Chongqing University Cancer Hospital, Chongqing 4000430, China; f Department of Orthopaedic Surgery, Beijing Hospital, Chinese Academy of Medical Sciences & Peking Union Medical College, Beijing 100730, China; g Department of Orthopaedic Surgery, Wuxi Hospital Affiliated to Nanjing University of Chinese Medicine, Wuxi, Jiangsu 214071, China; h Department of Orthopaedic Surgery, Benq Medical Center, The Affiliated Benq Hospital of Nanjing Medical University, Nanjing, Jiangsu 210019, China; i Department of Orthopaedic Surgery, Yibin Second People's Hospital, Affiliated with West China School of Medicine, Yibin, Sichuan 644000, China; j Department of Orthopedics, Jiangxi Hospital of Traditional Chinese Medicine, Jiangxi University of Traditional Chinese Medicine, Nanchang, Jiangxi 330006, China; k Department of Oncology, The Affiliated Hospital of Weifang Medical University, Weifang, Shandong 261053, China; m Stem Cell Biology and Therapy Laboratory of the Pediatric Research Institute, The National Clinical Research Center for Child Health and Disorders, and Ministry of Education Key Laboratory of Child Development and Disorders, the Children's Hospital of Chongqing Medical University, Chongqing 400016, China; n Division of Research and Development, Decoding Therapeutics, Inc., Mt Prospect, IL 60056, USA; o Laboratory of Craniofacial Biology and Development, Department of Surgery Section of Plastic Surgery, The University of Chicago Medical Center, Chicago, IL 60637, USA

**Keywords:** GAPDH, Gene therapy, Oxidative stress, Packaging cell line, Reactive oxygen species, Recombinant adenovirus

## Abstract

Recombinant adenovirus (rAdV) is a commonly used vector system for gene transfer. Efficient initial packaging and subsequent production of rAdV remains time-consuming and labor-intensive, possibly attributable to rAdV infection-associated oxidative stress and reactive oxygen species (ROS) production. Here, we show that exogenous GAPDH expression mitigates adenovirus-induced ROS-associated apoptosis in HEK293 cells, and expedites adenovirus production. By stably overexpressing GAPDH in HEK293 (293G) and 293pTP (293GP) cells, respectively, we demonstrated that rAdV-induced ROS production and cell apoptosis were significantly suppressed in 293G and 293GP cells. Transfection of 293G cells with adenoviral plasmid pAd-G2Luc yielded much higher titers of Ad-G2Luc at day 7 than that in HEK293 cells. Similarly, Ad-G2Luc was amplified more efficiently in 293G than in HEK293 cells. We further showed that transfection of 293GP cells with pAd-G2Luc produced much higher titers of Ad-G2Luc at day 5 than that of 293pTP cells. 293GP cells amplified the Ad-G2Luc much more efficiently than 293pTP cells, indicating that exogenous GAPDH can further augment pTP-enhanced adenovirus production. These results demonstrate that exogenous GAPDH can effectively suppress adenovirus-induced ROS and thus accelerate adenovirus production. Therefore, the engineered 293GP cells represent a superfast rAdV production system for adenovirus-based gene transfer and gene therapy.

## Introduction

Human adenovirus serotype 5 (Ad5) is a linear 36 kb double-stranded, nonenveloped DNA virus in the shape of a 70–90 nm icosahedral particle with an outer protein shell surrounding an inner nucleoprotein core.^1^, ^2^, ^3^ Recombinant replication-deficient adenovirus (rAdV) represents one of the most commonly used viral vectors for *in vitro* and *in vivo* gene delivery.^1^, ^2^, ^3^, ^4^, ^5^, ^6^ rAdV has several attractive features for gene transfer including large cargo capacity, easy preparation of high titer viral stocks, high levels of transgene expression, and the ability to transduce a wide range of mammalian tissues and dividing/non-dividing cells.^3^^,^^5^^,^^6^ Furthermore, adenovirus-mediated gene delivery is transient and does not involve DNA integration into the host genome so insertional mutagenesis rarely occurs.^2^^,^^3^^,^^5^ Lastly, adenovirus DNA replication and adenovirus life cycle have been well understood and studied, which further benefits the utility of recombinant adenoviruses.^1^, ^2^, ^3^^,^^5^ In addition to its applications in gene transfer and gene therapy, adenoviral vectors are popular choices for oncolytic viral therapy, cancer immunotherapy, and DNA/RNA-based vaccination.^7^, ^8^, ^9^, ^10^

Since rAdV is void of the critical E1 viral genes, rendering rAdV replication-deficient and creating package capacity for transgenes of interest, the production of AdV requires the use of packaging cells, such as the commonly used HEK293 cells.^3^ HEK293 cells were generated by transforming human embryonic kidney (HEK) cells with sheared adenoviral genomic DNA containing the Ad5 sequences between 1 and 4137 nt.^11^^,^^12^ For the past decades, several technical advances have allowed us to easily introduce transgene fragments into adenoviral genome.^13^, ^14^, ^15^, ^16^, ^17^, ^18^, ^19^ We also made significant efforts in facilitating rAdV production by establishing more efficient packaging HEK293 derivative 293pTP and RAPA lines.^20^^,^^21^ Nonetheless, the initial packaging and production of rAdV still requires 10–14 days, and thus remains a time-consuming and labor-intensive process.

Adenovirus production is a highly dynamic and well-orchestrated cellular process of coordinated temporal viral gene expression, efficient adenoviral DNA replication, and adequate synthesis of viral capsid proteins for adenovirus virion assembly.^1^^,^^22^ Meanwhile, the newly generated rAdVs exert a profound cytopathic effect on host cells.^23^^,^^24^ It is well established that viral infection often induces mitochondria-associated components to boost the production of reactive oxygen species (ROS), which subsequently leads to ROS-associated cell apoptosis.^25^, ^26^, ^27^ Furthermore, it has been reported that adenovirus-induced cell death is associated with changes in metabolic profiles of the infected cells, in which adenovirus-induced metabolomic changes precede cell death in most cases.^28^ Therefore, it is conceivable that suppression of adenovirus-induced ROS production and associated apoptosis may significantly expedite rAdV production in HEK293 packaging cells.

Commonly known as a housekeeping gene, glyceraldehyde 3-phosphate dehydrogenase (GAPDH) is a 37 kDa enzyme (EC 1.2.1.12) that catalyzes the sixth step of glycolysis responsible for glucose breakdown to provide energy and carbon sources.^29^^,^^30^ Increasing evidence indicates that, in addition to its metabolic function, GAPDH plays important roles in many other cellular processes including endoplasmic reticulum to Golgi transport, transcription activation, participation in protein–protein interactions, adhesion to extracellular matrix, and association with neurodegeneration and cancer.^29^, ^30^, ^31^, ^32^ In fact, it has been well recognized that GAPDH is one of the most prominent cellular targets of oxidative modifications when ROS and reactive nitrogen species are formed during metabolism and under stress conditions,^33^ suggesting that GAPDH may play a pivotal role in regulating cellular redox.^34^

In this study, we sought to investigate whether exogenous GAPDH expression would alleviate adenovirus-induced oxidative stress and ROS-associated apoptosis in HEK293 cells, and subsequently expedite adenovirus production. By stably overexpressing GAPDH in HEK293 and 293pTP cells (designated as 293G and 293GP, respectively), we demonstrated that rAdV infection induced profound ROS production and cell apoptosis in the parental lines, which was significantly diminished in 293G and 293GP cells, respectively. We demonstrated that transfection of 293G cells with adenoviral plasmid pAd-G2Luc yielded much higher titers of Ad-G2Luc at as early as day 7 than that in HEK293 cells. Similarly, Ad-G2Luc was amplified more efficiently in 293G than in HEK293 cells. We further showed that transfection of 293GP cells produced much higher titers of Ad-G2Luc at as early as day 5 than that of 293pTP cells. It was shown that 293GP cells amplified the Ad-G2Luc much more efficiently than 293pTP cells, confirming that exogenous GAPDH can further augment pTP-enhanced adenovirus production. Collectively, our results demonstrate that exogenous GAPDH can effectively suppress adenovirus-induced oxidative stress and ROS production, and subsequently accelerate adenovirus production. Thus, the established 293GP cells that overexpress both GAPDH and Ad5-pTP in HEK293 cells represent one of the fastest rAdV production systems for adenovirus-based gene transfer and gene therapy.

## Materials and methods

### Cell culture and chemicals

Human HEK293 (or 293) and human bladder cancer line T24 cells were purchased from the American Type Culture Collection (Manassas, VA, USA). HEK293-derived 293pTP cells were previously described.^20^ Human urine stem cells (USCs) were isolated as previously described.^35^ The iMEFs cells were immortalized mouse embryonic fibroblasts as previously described.^36^, ^37^, ^38^ All cells were maintained in complete Dulbecco's modified Eagle medium (DMEM) supplemented with 10% fetal bovine serum (Gemini Bio-Products), 100 U/mL penicillin, and 100 μg/mL streptomycin at 37 °C in 5% CO_2_ as described.^39^, ^40^, ^41^, ^42^, ^43^, ^44^, ^45^ Restriction endonuclease enzymes, M-MuLV reverse transcriptases, and deoxynucleoside triphosphates (dNTPs) were purchased from New England Biolabs (Ipswich, MA, USA) and GenScript USA Inc (Catalog #C01581-10; Piscataway, NJ, USA), respectively. Unless indicated otherwise, all chemicals were purchased from Sigma–Aldrich (St Louis, MO, USA) or Fisher Scientific (Pittsburgh, PA, USA).

### Construction of retroviral GAPDH expression vector and establishment of the GAPDH stable expression lines, 293G and 293GP, from HEK-293 and 293pTP cells, respectively

The coding region of human GAPDH was PCR amplified and subcloned into our homemade retroviral expression vector pSEPI 46, 47, 48, 49 at the *Bam*H1/*Mlu*I sites, resulting in pSEPI-GAPDH (Fig. S1A). The construct was verified by DNA sequencing. Retrovirus RV-GAPDH was packaged by co-transfecting pSEPI-GAPDH, pCL-Ampho, and pCMV-VSVG into HEK-293 cells as previously reported.^36^^,^^50^^,^^51^ The packaged retrovirus supernatants were collected at 36 h, 48 h, 60 h, and 72 h after transfection, filtered, and stored at 4 °C or −80 °C before use.

For generating stable GAPDH expression lines, exponentially growing HEK-293 and 293pTP cells were infected with the packaged RV-GAPDH as previously described.^49^^,^^50^^,^^52^ The infected cells were subjected to three rounds of puromycin selection (final concentration at 0.5 μg/mL). The resultant stable lines were designated as 293G and 293 GP lines, respectively (Fig. S1B). The exogenous GAPDH expression in the stable lines was verified by touchdown-quantitative real-time PCR (TqPCR) analysis (Fig. S1C). Aliquots of the stable lines were kept in LN_2_ tanks for long-term storage.

### Construction, packaging, amplification, and infection of recombinant adenoviruses Ad-G2Luc and Ad-RFP

Recombinant adenoviruses were generated using the AdEasy system as described.^3^^,^^14^^,^^15^ Briefly, the coding regions of Gaussia luciferase (GLuc) and copGFP, separated by the self-cleaving peptide E2A, were PCR amplified and cloned into an adenoviral shuttle vector. The linearized shuttle vector was used for homologous recombination reactions with the adenoviral backbone vector pAdEasy1 in BJ5183 cells to generate the recombinant adenoviral vector pAd-G2Luc. Except for the packaging experiments reported here, Ad-G2Luc adenovirus was packaged in 293pTP cells, and amplified to high titers in HEK-293, 293pTP, or RAPA cells.^20^^,^^21^ The Ad-G2Luc expresses both functional GLuc and copGFP, which is thus ideal for both qualitative and quantitative analyses as previously described.^53^, ^54^, ^55^, ^56^ The control adenovirus Ad-RFP was constructed using the Gibson DNA Assembly-based OSCA system as described.^19^

Adenovirus packaging was carried out as described.^15^^,^^57^^,^^58^ Briefly, exponentially growing HEK293, 293G, 293pTP, or 293GP cells were seeded in 100 mm cell culture dishes at ∼30% density for 4 h. Once the cells were attached, 10 μg of PacI-cut pAd-G2Luc were mixed with PEI transfection agent (Polysciences, Inc., Warrington, PA, USA) and added to the cells maintained in serum-free condition, and incubated at a 37 °C 5% CO_2_ incubator for 4 h. The DNA/PEI mix was then replaced with complete DMEM. Adenoviral lysates were collected on day 7 (for HEK293 and 293G) and day 5 (for 293pTP and 293GP) after transfection.

To assess the genuine titer differences among the adenoviral lysates produced from different packaging lines, all adenoviral infection experiments were carried out in the absence of polybrene, as we previously reported that polybrene can drastically enhance adenoviral transduction efficiency.^59^

### Total RNA isolation and TqPCR analysis

Total RNA isolation was conducted according to the method previously described.^60^, ^61^, ^62^, ^63^ Briefly, subconfluent HEK293, 293G, 293pTP, and 293GP were lyzed using the NucleoZOL reagent kit (Takara Bio USA, San Jose, CA) by following the manufacturer's instructions. Reverse transcription reactions were performed using hexamers and M-MuLV Reverse Transcriptase (NEB) to obtain cDNA products as PCR templates. Human GAPDH-specific qPCR primers were designed using the Primer3Plus program (Table S1). TqPCR was performed on a CFX-Connect instrument (Bio-Rad Laboratories, Hercules, CA) using 2 × Biotium Forget-Me-Not™ EvaGreen qPCR Master Mix (Biotium, Inc., Fremont, CA) as described.^64^, ^65^, ^66^ All TqPCR reactions were performed in triplicate. Human *TBP* (TATA-box binding protein) and *HPRT1* (hypoxanthine phosphoribosyltransferase 1) were internal references. The 2^−ΔΔCq^ method was used for quantification analysis of gene expression levels, as described.^67^, ^68^, ^69^, ^70^

### Adenovirus-induced ROS generation and detection

Subconfluent HEK293, 293G, 293pTP, and 293GP cells were seeded in 35 mm cell culture dishes and infected with Ad-RFP at the MOI (multiplicity of infection) of 10 for 24 h. The infected cells were then incubated with the ROS sensor H2DCFDA (ThermoFisher; final concentration at 5 μM) at 37 °C for 30 min under dark condition, followed by the removal of the complete DMEM culture medium. For each dish, 3 mL of phosphate-buffered saline solution (PBS) was added, and the GFP (green fluorescent protein) and RFP (red fluorescent protein) signals were immediately examined and documented under a fluorescence microscope. Each assay condition was done in triplicate.

### Cell apoptosis analysis by flow cytometry

Cell apoptosis analysis was performed quantitatively using flow cytometry as previously described.^71^, ^72^, ^73^, ^74^ Briefly, exponentially growing HEK293, 293pTP, 293G, and 293 GP cells were seeded in 6-well cell culture plates at 1 × 10^5^ cells/well for 24 h, and then incubated with hydrogen peroxide (H_2_O_2_) at concentrations ranging from 4 to 9 μM for 6 h. The treated cells were trypsinized, collected (combined with the floater cells in the culture medium), and washed with PBS twice. The recovered cells were resuspended in 500 μL of PBS and stained with annexin V-FITC and propidium iodide (PI) (BioLegend, San Diego, CA) as previously described,^75^^,^^76^ followed by flow cytometry analysis using a NovoCyte Quanteon flow cytometer. Data analysis was done using Novoexpress software.

### Cell cycle analysis by flow cytometry

Cell cycle analysis was performed quantitatively using flow cytometry as previously described.^51^^,^^77^^,^^78^ Briefly, exponentially growing HEK293, 293G, 293pTP, and 293 GP cells were seeded in 6-well cell culture plates at 10^5^ cells/well for 24 h, and then incubated with H_2_O_2_ at 5.0 μM for 6 h. The treated cells were trypsinized, collected, washed twice with PBS, and then fixed with 500 μL of pre-chilled 70% ethanol at 4 °C overnight. After fixation, the cells were incubated with the PI staining solution, containing RNase A (200 μg/mL), PI (50 μg/mL), and Triton X-100 (0.1% v/v) in PBS, at 4 °C for 30 min. After the excessive staining solution was washed off with PBS, the stained cells were subjected to NovoCyte Quanteon flow cytometry analysis. Cell cycle profiles were analyzed using the Novoexpress software.

### Adenovirus titer determination

In this study, the same percentage of adenoviral lysates collected from either transfection (for packaging) or infection (for amplification) samples was used for the side-by-side comparison experiments. The actual adenovirus titers were determined as previously described.^15^^,^^37^^,^^79^, ^80^, ^81^ Briefly, adenoviral lysates or high titer stocks were subjected to 5-fold or 10-fold serial dilutions, and then used to infect subconfluent HEK293 cells seeded in 6-weel cell culture plates. Since Ad-G2Luc expresses the copGFP marker, GFP^+^ cells were enumerated at 36 h post-infection. Adenovirus titers were calculated by averaging numbers of GFP^+^ cells for multiple dilutions for each lysate or virus stock and expressed in infectious units (IU) per mL (*i.e.*, IU/mL).

### Gaussia luciferase (GLuc) activity assay

The secreted GLuc activity assay was carried out as previously described.^53^^,^^55^^,^^82^^,^^83^ Specifically, the Ad-G2Luc-containing viral lysates were used to infect the cell lines of interest. At the indicated time points after infection, a volume of 50 μL was taken from the culture medium of each sample for the GLuc activity assay using the Secrete-Pair Gaussia Luciferase Assay Kit (Cat#LF062, GeneCopoeia, Rockville, MD). Each assay condition was carried out in triplicate.

### Data presentation and statistical analysis

All quantitative experiments were conducted in triplicate and/or repeated in three independent batches. Data were expressed as mean ± standard deviation. Statistical significances between groups were determined by one-way analysis of variance (ANOVA) and the student's *t*-test. A *P*-value <0.05 was considered statistically significant.

## Results

### Adenovirus infection induces a marked production of ROS that can be effectively mitigated by exogenously overexpressed GAPDH

**Figure 1:**
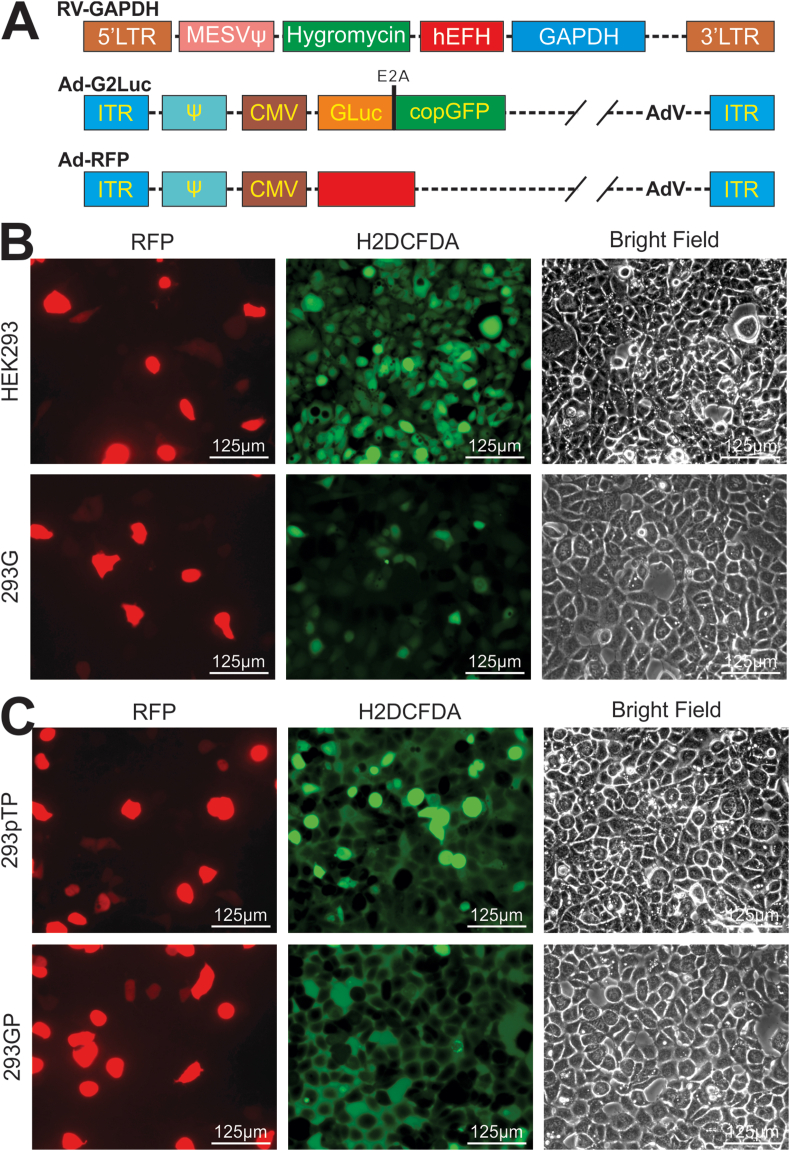
Exogenously expressed GAPDH effectively curtails adenovirus-induced production of reactive oxidative species (ROS) in the adenovirus packaging cells. **(A)** Schematic representation of a retroviral vector, RV-GAPDH, for stable GAPDH expression, a tester adenoviral vector, Ad-G2Luc that expresses an E2A-self-cleaved fusion protein of the secreted Gaussia luciferase (GLuc) and copGFP fluorescent protein, and a control adenoviral vector, Ad-RFP. The overall experimental design is illustrated in Figure S1. **(B, C)** Subconfluent HEK293 and 293G cells (B) or 293pTP and 293 GP cells (C) were infected with the same titer of Ad-RFP for 36 h, and ROS biosensor H2DCFDA (final concentration at 5 μM) was added to the infected cells for 30 min, followed by RFP and GFP imaging. Bright-field images were also recorded under transmissive light. Representative images are shown.

We first constructed the retroviral vector pSEHI-GAPDH that renders stable GAPDH overexpression in HEK293 and 293pTP cells (Fig. 1A; Fig. S1A). To facilitate tracking the effect of GAPDH overexpression on ROS production and adenovirus packaging, we also constructed a tester adenoviral vector, Ad-G2Luc that expresses an E2A-self cleaved fusion protein of the secreted Gaussia luciferase (GLuc) and copGFP fluorescent protein, as well as a control adenoviral vector, Ad-RFP (Fig. 1A). Two stable GAPDH expression lines, 293G and 293GP, were established using the packaged RV-GAPDH retrovirus as described (Fig. S1B).^50^^,^^71^^,^^84^, ^85^, ^86^ The exogenous expression levels of GAPDH in both stable lines were confirmed using TqPCR analysis (Fig. S1C).

To determine whether adenovirus infection induced ROS production, we infected subconfluent HEK293, 293G cells, 293pTP, and 293 GP cells with the same titer of Ad-RFP for 36 h, and then treated the infected cells with 5 μM ROS biosensor H2DCFDA for 30 min, followed by RFP and GFP imaging. We found significantly higher GFP signal (from H2DCFDA) was observed in HEK293 and 293pTP cells, compared with that in 293G and 293GP, respectively (Fig. 1B, C; Fig. S2A, B). These results demonstrate that adenovirus infection can induce a pronounced ROS generation, which can be effectively curtailed by exogenous GAPDH overexpression.

### Exogenously expressed GAPDH suppresses ROS-induced apoptosis in adenovirus packaging cells

**Figure 2:**
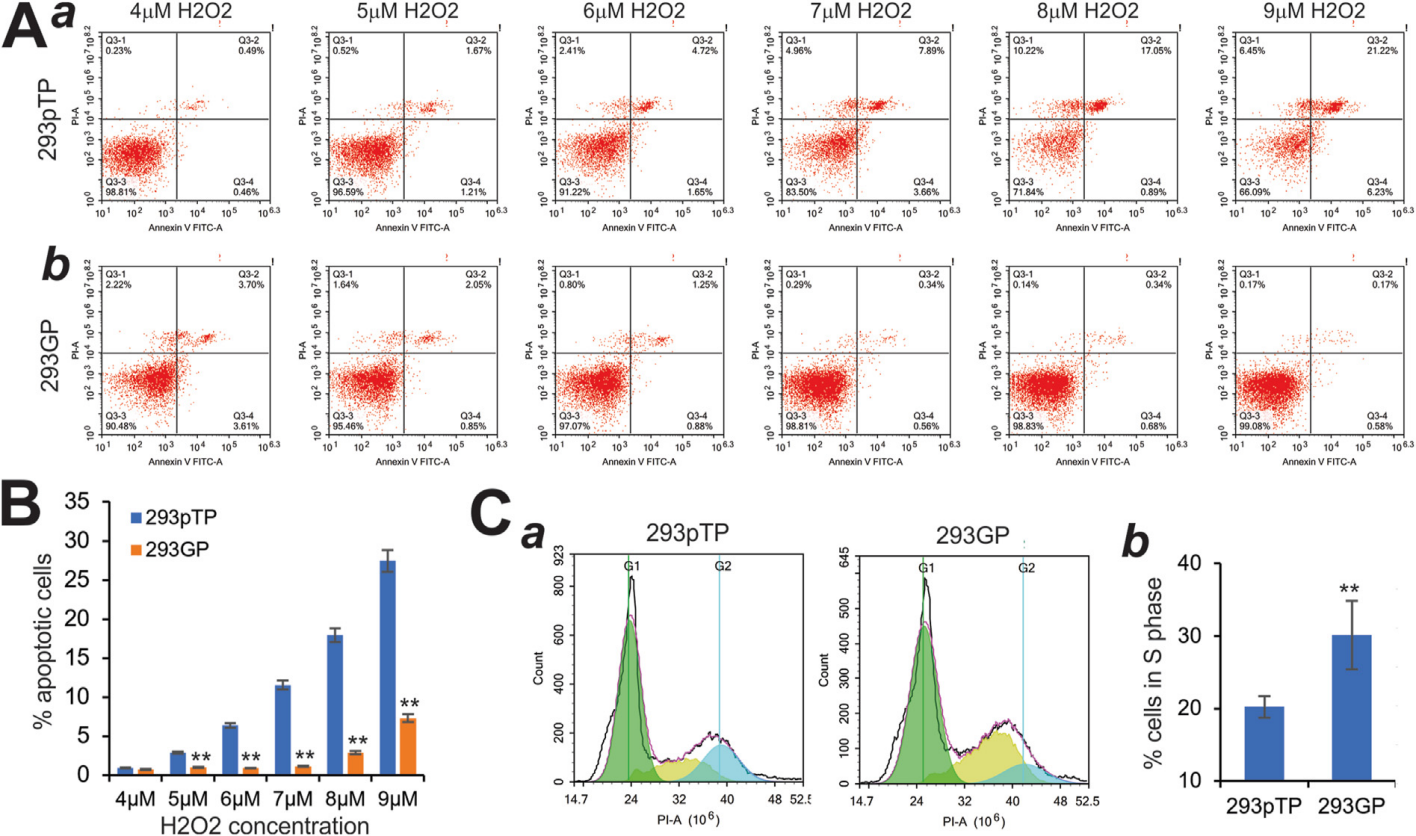
Exogenously expressed GAPDH suppresses reactive oxidative species (ROS)-induced apoptosis in the adenovirus packaging cells. **(A)** Subconfluent 293pTP **(*a*)** and 293GP **(*b*)** cells were treated with hydrogen peroxide (H_2_O_2_) at the indicated concentrations for 6 h. Cells were collected and stained with annexin V-FITC/PI for flow cytometric assay. Representative images are shown. **(B)** The percentage of apoptotic cells was calculated for 293pTP and 293GP cells treated with various concentrations of H_2_O_2_ treatment. ^∗∗^*P* < 0.01, compared with that of the 293pTP group. **(C)** Subconfluent 293pTP and 293GP cells were treated with H_2_O_2_ at 5 μM for 6 h. The treated cells were collected and subjected to flow cytometric cell cycle analysis **(*a*)**, and % cells in the S phase were calculated **(*b*)**. ^∗∗^*P* < 0.01, compared with that of the 293pTP group. Representative images are shown.

To quantitatively assess the anti-ROS effect exerted by the exogenously overexpressed GAPDH, we treated the subconfluent 293pTP and 293GP cells with varied concentrations of H_2_O_2_ for 6 h and collected the treated cells for apoptosis analysis. Our results revealed that H_2_O_2_ induced apparent apoptosis in 293pTP cells and that was effectively suppressed in 293GP cells (Fig. 2A, panels *a*, *b*). The quantitative analysis showed that H_2_O_2_ induced apoptosis in 293pTP cells in a concentration-dependent fashion, while the percentage of apoptotic cells was dramatically reduced in 293GP cells (*P* < 0.01) (Fig. 2B). Furthermore, we analyzed the cell cycle profiles of the 293pTP and 293GP cells treated with 5 μM H_2_O_2_ (Fig. 2C, panel *a*), and found that the percentage of cells in S phase was significantly reduced in 293pTP cells but was significantly restored in the GAPDH overexpressing 293GP cells (Fig. 2C, panel *b*). Collectively, these results demonstrate that overexpression of GAPDH can effectively suppress adenovirus-induced ROS production and ROS-associated apoptosis.

### Exogenous GAPDH expression accelerates the initial packaging and amplification of recombinant adenovirus in 293G cells

**Figure 3:**
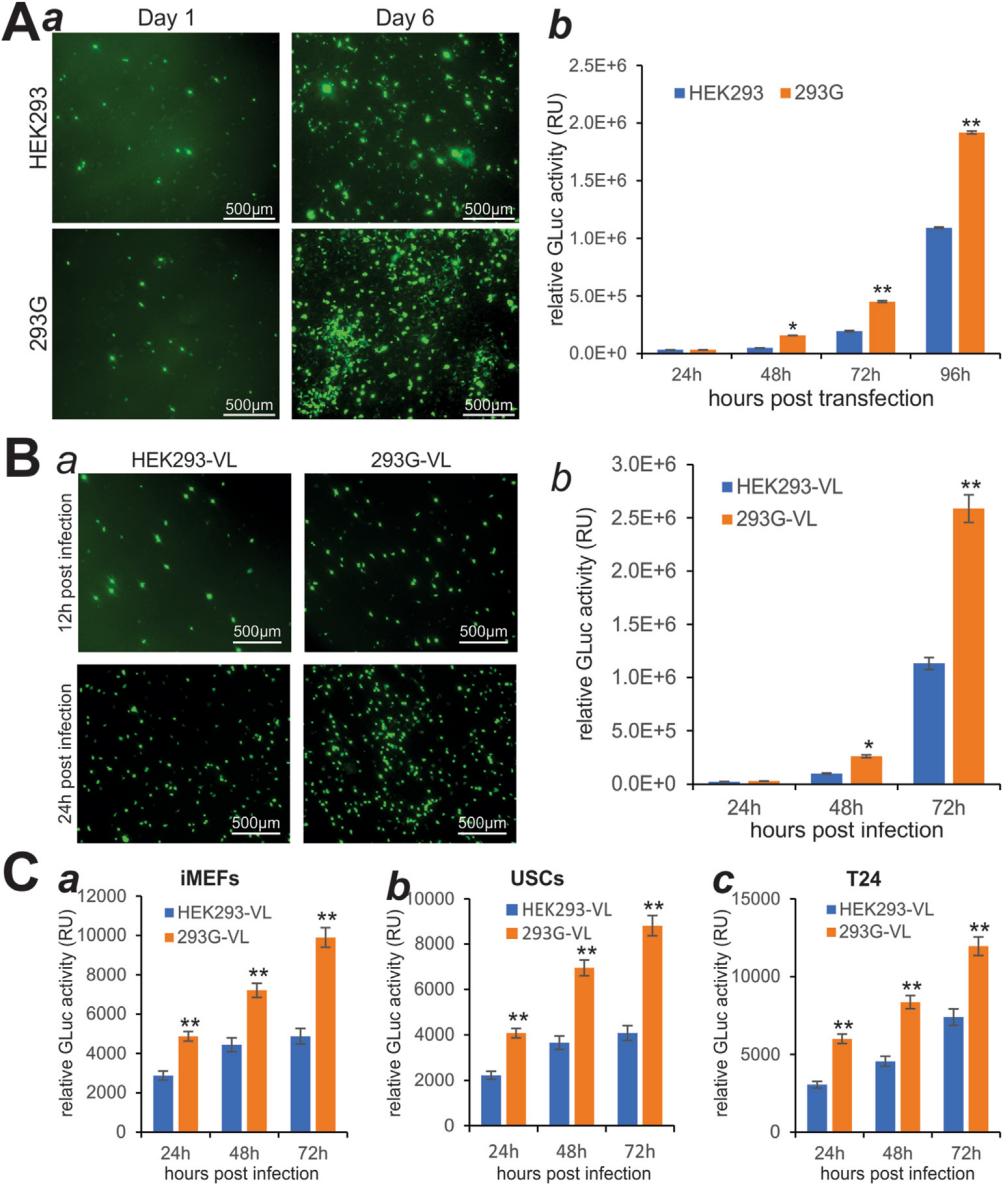
Exogenous GAPDH expression in HEK293 cells accelerates the packaging of recombinant adenovirus. **(A)** Subconfluent HEK293 and 293G cells were transfected with pAd-G2Luc plasmid. GFP signal was recorded on days 1 and 6 after transfection **(*a*)**, while GLuc activities were quantitatively assessed at the indicated time points **(*b*)**. ^∗^*P* < 0.05, ^∗∗^*P* < 0.01, compared with that of the HEK293 group. **(B)** An equal percentage of the viral lysate (VL) prepared from HEK293 (*i.e.*, HEK293-VL) and 293G (*i.e.*, 293G-VL) (A) was used to infect HEK293 cells. GFP signal was recorded at 12 h and 24 h after infection **(*a*)**, while GLuc activities were measured at the indicated time points after infection **(*b*)**. ^∗^*P* < 0.05, ^∗∗^*P* < 0.01, compared with that of the HEK293-VL group. **(C)** HEK293-VL and 293G-VL were used to infect non-packaging cells including iMEFs **(*a*)**, USCs **(*b*)**, and T24 cells **(*c*)**. GLuc activities were determined at the indicated time points. ^∗∗^*P* < 0.01, compared with that of the HEK293-VL group.

We next investigated whether GAPDH overexpression in HEK293 cells affected the adenovirus packaging efficiency. When subconfluent HEK293 and 293G cells were transfected with the same amount of PacI-linearized pAd-G2Luc plasmid, the GFP signal was markedly higher in 293G cells than in HEK293 cells on day 6, while the GFP signal was detected at a similar level on day 1 in both cell lines (Fig. 3A, panel *a*). Quantitative GLuc activity analysis revealed that except at 24 h post-transfection GLuc activities were significantly higher in 293G cells than in HEK293 cells at 48 h, 72 h, and 96 h post-transfection (Fig. 3A, panel *b*), indicating that 293G packaged Ad-G2Luc more efficiently than that in the parental HEK293 cells.

To determine titer differences of the packaged Ad-G2Luc, we utilized 5% of the harvested viral lysates produced from HEK293 (*i.e.*, HEK293-VL) and 293G (*i.e.*, 293G-VL) to infect HEK293, and found that the GFP signal was significantly higher in the 293G-VL infected cells at 24 h than that in HEK293-VL infected cells (Fig. 3B, panel *a*). Higher GLuc activities of 293G-LV were also confirmed by quantitative analysis of GLuc activities of the infected cells at multiple time points (Fig. 3B, panel *b*). Furthermore, we used 10% of HEK293-VL and 293G-VL to infect three non-packaging cells, iMEFs, USCs, and T24, and quantitative GLuc activity analysis revealed that 293G-VL infected cells always yielded significantly higher GLuc activities than that of HEK293-VL infected iMEFs, USCs, and T24 cells (Fig. 3C, panels *a*–*c*). Collectively, these results demonstrate that GAPDH-overexpressing 293G can package Ad-G2Luc virus with much higher efficiency.

**Figure 4:**
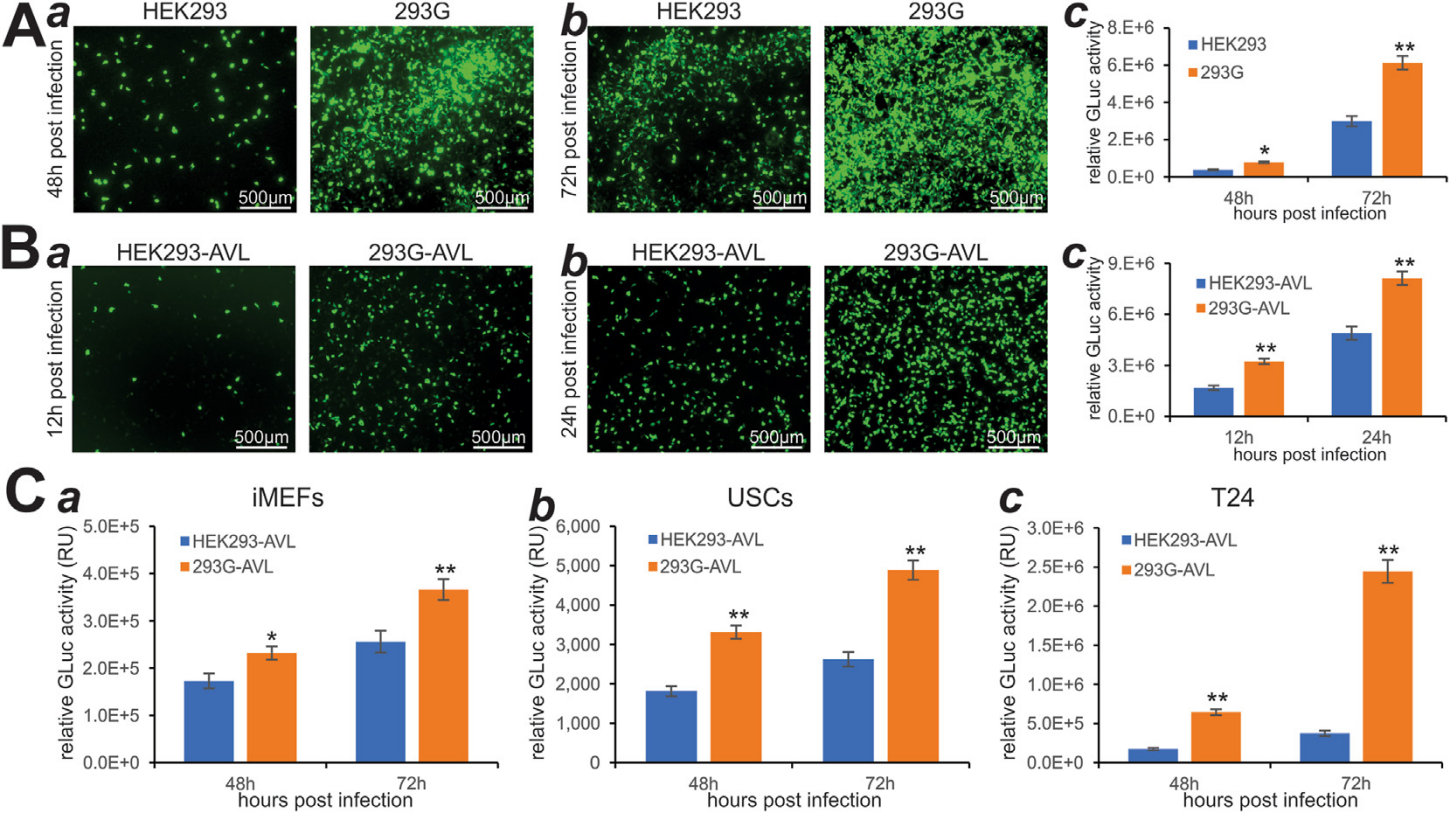
Exogenous GAPDH expression in HEK293 cells enhances the amplification and production of recombinant adenovirus. **(A)** Subconfluent HEK293 and 293G cells were infected with the same titter of Ad-G2Luc. GFP signal was recorded at 48 h **(*a*)** and 72 h **(*b*)** after infection, while GLuc activities were quantitatively assessed at 48 h and 72 h after infection **(*c*)**. ^∗^*P* < 0.05, ^∗∗^*P* < 0.01, compared with that of the HEK293 group. **(B)** An equal percentage of the amplification viral lysate (AVL) prepared from HEK293 (*i.e.*, HEK293-AVL) and 293G (*i.e.*, 293G-AVL) was used to infect HEK293 cells. GFP signal was recorded at 12 h **(*a*)** and 24 h **(*b*)** after infection, whereas GLuc activities were measured at 12 h and 24 h after infection (***c***). ^∗∗^*P* < 0.01, compared with that of the HEK293-AVL group. **(C)** HEK293-AVL and 293G-AVL were used to infect subconfluent iMEFs **(*a*)**, USCs **(*b*)**, and T24 cells **(*c*)**. GLuc activities were determined at the indicated time points, while the GFP signal was much weaker as shown in Figure S3. ^∗^*P* < 0.05, ^∗∗^*P* < 0.01, compared with that of the HEK293-AVL group.

We further analyzed the effect of exogenous GAPDH on adenovirus amplification. Experimentally, we infected subconfluent HEK293 and 293G with titered Ad-G2Luc at the MOI of 20 and found that the numbers of GFP^+^ cells were drastically higher in 293G cells, compared with that in HEK293 cells (Fig. 4A, panels *a*, *b*), which was further confirmed by quantitative GLuc activity analysis (Fig. 4A, panels *a*, *b*). We used 5% of the amplified virus lysate (AVL) from HEK293 (HEK293-AVL) and 293G (293G-AVL) to infect subconfluent HEK293 cells and found that the numbers of GFP^+^ cells were significantly higher for 293G-AVL than that for HEK293-AVL at 12 h and 24 h post-infection (Fig. 4B, panels *a*, *b*), which was also confirmed by the quantitative GLuc analysis (Fig. 4B, panel *c*). Lastly, we used 10% of HEK293-AVL and 293G-AVL to infect iMEFs, USCs, and T24 and found that higher numbers of GFP^+^ cells were observed in 293G-AVL infected T24 cells, to a much lesser extent, iMEF cells (Fig. S3A, B), although no GFP signal was detected in USCs (data not shown). Nonetheless, GLuc activity analysis revealed that 293G-AVL infected cells always yielded significantly higher GLuc activities than that of HEK293-AVL infected iMEFs, USCs, and T24 cells (Fig. 4C, panels *a*–***c***). Taken together, the above findings demonstrate that GAPDH overexpression can significantly promote adenovirus packaging and amplification efficiency in HEK293 cells.

### Exogenous GAPDH expression further augments pTP-accelerated adenovirus packaging and amplification efficiency in 293GP cells

**Figure 5:**
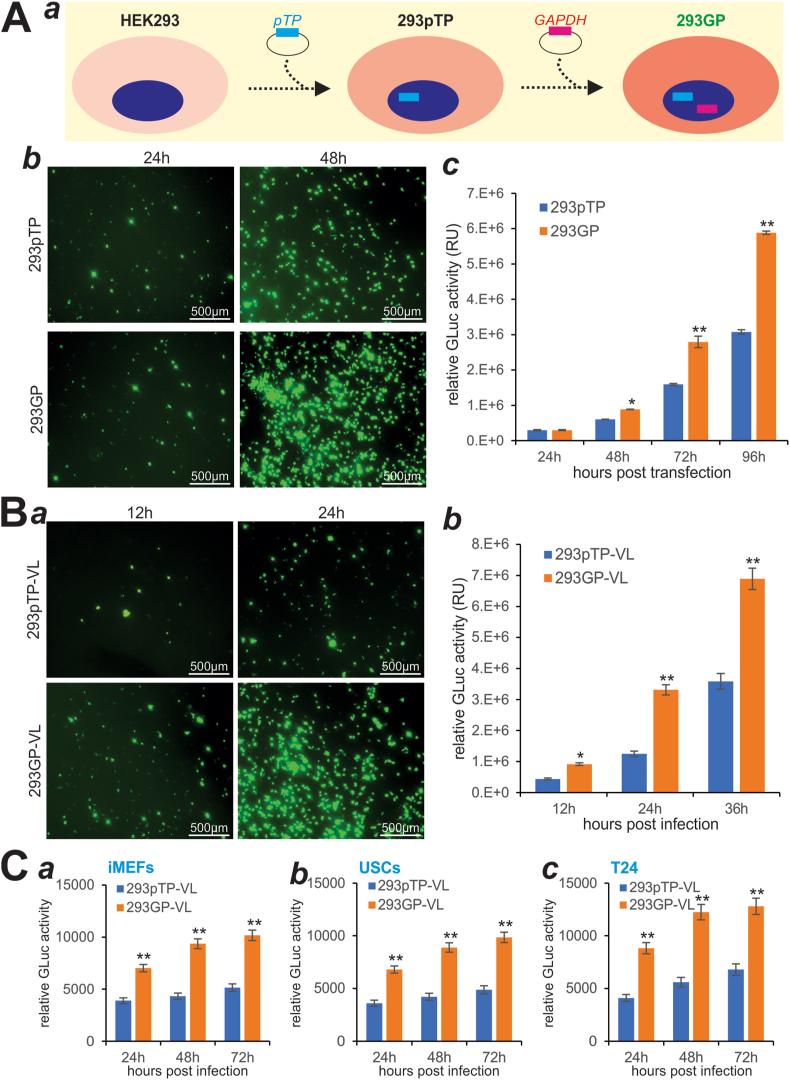
Exogenous GAPDH expression further potentiates pTP-accelerated packaging of recombinant adenovirus. **(A)** Schematic representation of the establishment of 293GP cells. RV-GAPDH was used to infect 293pTP cells that were previously established **(*a*)**. Subconfluent 293pTP and 293GP cells were transfected with pAd-G2Luc plasmid. GFP signal was recorded at 24 h and 48 h after transfection **(*b*)**, while GLuc activities were quantitatively assessed at the indicated time points **(*c*)**. ^∗^*P* < 0.05, ^∗∗^*P* < 0.01, compared with that of the HEK293 group. **(B)** An equal percentage of the viral lysate (VL) prepared from 293pTP (*i.e.*, 293pTP-VL) and 293GP (*i.e.*, 293GP-VL) was used to infect HEK293 cells. GFP signal was recorded at 12 h and 24 h after infection **(*a*)**, while GLuc activities were measured at the indicated time points after infection **(*b*)**. ^∗^*P* < 0.05, ^∗∗^*P* < 0.01, compared with that of the 293pTP-VL group. **(C)** 293pTP-VL and 293GP-VL were used to infect iMEFs **(*a*)**, USCs **(*b*)**, and T24 cells **(*c*)**. GLuc activities were determined at the indicated time points. ^∗∗^*P* < 0.01, compared with that of the 293pTP-VL group.

We previously demonstrated that overexpression of Ad5 pTP gene in HEK293 cells (293pTP) markedly accelerated adenovirus production.^20^ Here, we sought to test if GAPDH overexpression would further augment adenovirus production in 293pTP cells by stably transducing this cell line with packaged RV-GAPDH, resulting in 293GP cells (Fig. 5A, panel *a*). By transfecting the linearized pAd-G2Luc plasmid DNA into subconfluent 293pTP and 293GP cells, we found that significantly increased GFP^+^ cells were found in 293GP cells at 48 h after transfection, compared with that in 293pTP cells (Fig. 5A, panel *b*). Quantitative GLuc activity analysis showed that 293GP cells yielded much higher GLuc activities at 48 h, 72 h, and 96 h after transfection, compared with that of 293pTP cells (Fig. 5A, panel *c*).

When HEK293 cells were infected with the virus lysate retrieved from 293GP cells, 293GP-VL, much higher numbers of GFP^+^ cells and higher GLuc activities were observed at all tested time points, compared with that with 293pTP-VL (Fig. 5B, panels *a*, *b*). We also used 10% of 293pTP-VL and 293GP-VL to infect iMEFs, USCs, and T24 cells. Quantitative GLuc activity analysis revealed that 293GP-VL infected cells always yielded higher GLuc activities than that of 293pTP-VL infected iMEFs, USCs, and T24 cells (Fig. 5C, panels *a*–*c*). The above results demonstrate that GAPDH overexpression can further potentiate adenovirus packaging efficiency in 293GP cells.

**Figure 6:**
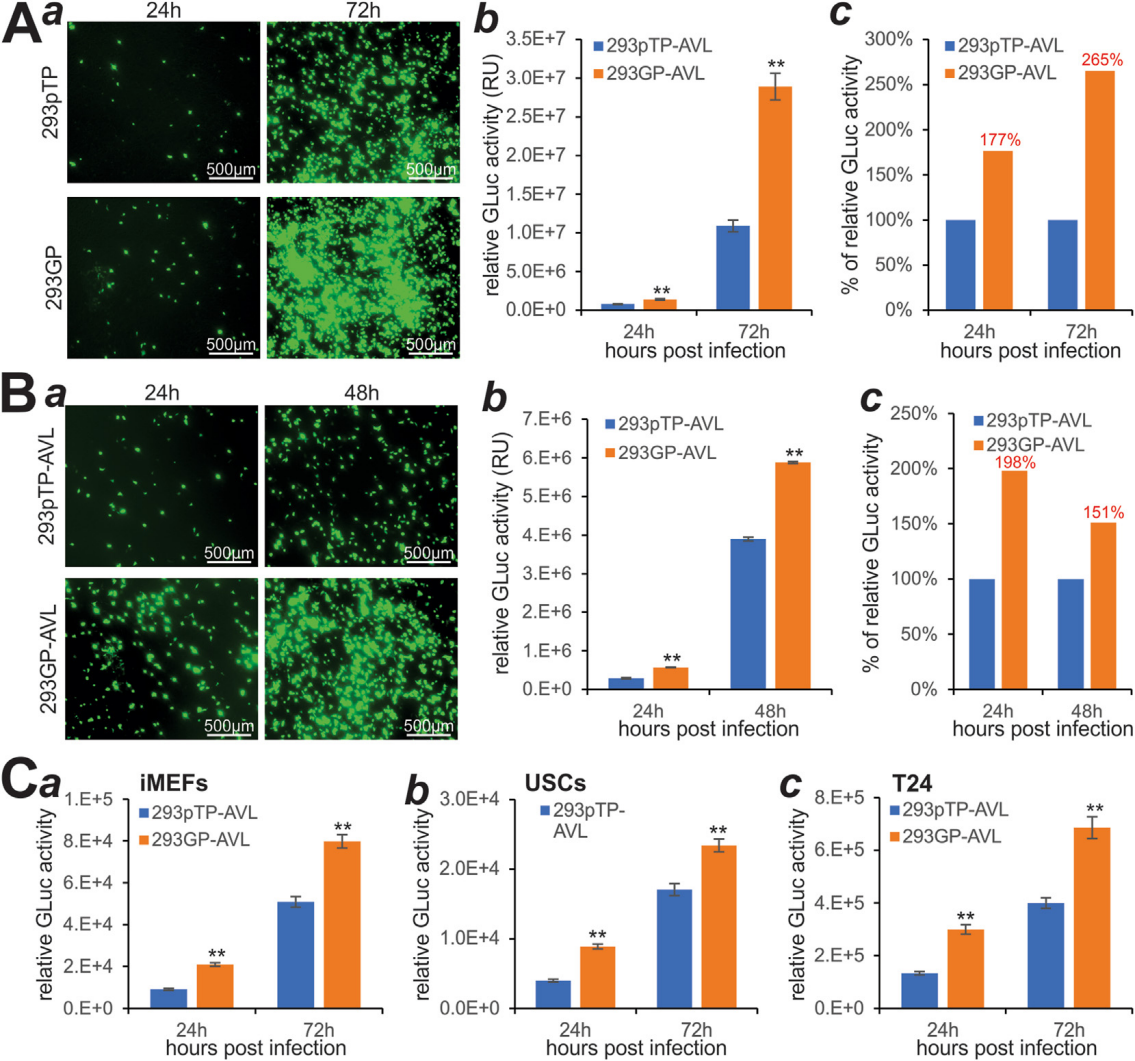
Exogenous GAPDH expression further augments pTP-enhanced amplification and production of recombinant adenovirus. **(A)** Subconfluent 293pTP and 293GP cells were infected with the same titter of Ad-G2Luc. GFP signal was recorded at 24 h and 72 h **(*a*)** after infection, while GLuc activities were quantitatively assessed at 24 h and 72 h after infection **(*b*, *c*)**. ^∗∗^*P* < 0.01, compared with that of the 293pTP group. **(B)** An equal percentage of the amplification viral lysate (AVL) prepared from 293pTP (*i.e.*, 293pTP-AVL) and 293GP (*i.e.*, 293GP-AVL) was used to infect HEK293 cells. GFP signal was recorded at 24 h and 48 h after infection **(*a*)**, whereas GLuc activities were measured at 24 h and 48 h after infection **(*b*, *c*)**. ^∗∗^*P* < 0.01, compared with that of the 293pTP-AVL group. **(C)** 293pTP-AVL and 293GP-AVL were used to infect subconfluent iMEFs **(*a*)**, USCs **(*b*)**, and T24 cells **(*c*)**. GLuc activities were determined at 24 h and 72 h after infection, while the GFP signal was much weaker as shown in Figure S4. ^∗∗^*P* < 0.01, compared with that of the 293pTP-AVL group.

We further compared the adenovirus amplification efficiency in 293pTP versus 293GP cells. We infected subconfluent 293pTP and 293GP with titered Ad-G2Luc at the MOI of 10 and found that the numbers of GFP^+^ cells were drastically higher in 293GP cells at 72 h post-infection, compared with that in 293pTP cells (Fig. 6A, panel *a*), which was further confirmed by quantitative GLuc activity analysis (Fig. 6A, panels *b*, *c*).

Using 5% of the amplified virus lysate 293pTP-AVL and 293GP-AVL, we infected subconfluent HEK293 cells and found that the numbers of GFP^+^ cells were significantly higher for 293GP-AVL than that for 293pTP-AVL at 24 h and 48 h post-infection (Fig. 6B, panel *a*), which was also confirmed by the quantitative GLuc analysis (Fig. 6B, panels *b*, *c*). Lastly, we used 10% of 293pTP-AVL and 293GP-AVL to infect iMEFs, USCs, and T24 cells and found that higher numbers of GFP^+^ cells were observed in 293GP-AVL infected T24 cells, to a much lesser extent, iMEF cells (Fig. S4A, B), while no GFP signal was detected in USCs (data not shown). Nonetheless, GLuc activity analysis revealed that 293GP-AVL infected cells always yielded significantly higher GLuc activities than that of 293pTP-AVL infected iMEFs, USCs, and T24 cells (Fig. 6C, panels *a*–*c*). Collectively, our findings strongly suggest that GAPDH overexpression, along with Ad5 pTP overexpression, in HEK293 cells may represent one of the most efficient adenovirus packaging and amplification systems for recombinant adenovirus production.

## Discussion

### Exogenous GAPDH expression facilitates adenovirus production by suppressing adenovirus-induced oxidative stress and ROS-associated apoptosis in HEK293 cells

It is well documented that adenovirus infection causes a profound cytopathic effect in host cells, initially attributed to adenovirus-induced host cell gene expression shutdown.^87^ However, in adenovirus-infected individuals, ROS were reportedly produced within minutes of Ad5 infection of macrophages and this oxidative stress supports Ad5-induced cytokine secretion.^25^ Since mitochondria-mediated oxidative stress is common during viral infection,^26^ here we demonstrated that adenovirus infection induced a remarkable ROS production in HEK293 and its derivative lines, suggesting that oxidative stress may undermine efficient rAdV production in the packaging cells.

Oxidative stress refers to elevated intracellular ROS levels that cause damage to lipids, proteins, and DNA.^88^ While oxidative stress has been linked to numerous pathologic conditions,^31^^,^^32^^,^^89^ ROS are signaling molecules for redox biology that maintain physiological functions.^90^ Redox homeostasis within the cell may be altered when the global shutdown of mitochondrial function under conditions of oxidative stress could contribute to apoptosis because of the dramatic decrease in cellular energy supply.^89^ As a key glycolytic enzyme and cellular energy provider, GAPDH is one of the most prominent cellular targets of post-translational modifications, and during metabolic and oxidative stress, serves as a target of different oxidative post-translational modifications, such as sulfenylation, S-thiolation, nitrosylation, and sulfhydration.^34^ Increasing evidence indicates that GAPDH serves as cytosolic thiol switches and regulates numerous cellular functions.^33^ It has been reported that GAPDH may play an oncogenic role in cancer through DNA copy number amplification, altered promoter methylation, and/or FOXM1-regulated overexpression.^31^^,^^32^ Our findings are consistent with the non-glycolytic function of GAPDH in redox regulation. It is conceivable that under metabolic and oxidative stresses GAPDH overexpression may act as cytosolic thiol switches and alleviate ROS-associated apoptosis in HEK293 cells, subsequently leading to more efficient adenovirus replication and packaging in 293G and 293GP cells.

### Exogenous expression of both GAPDH and Ad5 pTP in HEK293 cells represents one of the most efficient adenovirus production systems

Even though rAdV has become one of the most used gene delivery systems in basic, translational, and clinical research, efficiently packaging and producing rAdV remains a technical challenge and time-consuming process for many investigators.^1^^,^^3^^,^^5^ We previously demonstrated that overexpression of Ad5 terminal binding protein pTP (*i.e.*, 293pTP) can significantly facilitate the initial packaging and subsequent amplification processes of rAdV production.^20^ Ad5 pTP plays an essential role in viral DNA replication since DNA replication is initiated by covalent coupling of the dCMP residue of the 5′ termini inverted terminal repeats to pTP and Ad5 DNA Pol complex in 3′-OH group, which serves as a primer for further elongation by a strand displacement mechanism.^22^

More recently, we conducted a functional analysis of five human Ad5 viral genes, including E1A, E1B19K/55K, pTP, DBP, and DNA Pol, and host factor OCT1 (organic cation transporter 1), and through different combinations delineated their contributions to adenovirus production and amplification.^21^ Using our optimized *piggyBac* transposon-based gene expression system,^37^^,^^46^^,^^91^ which can effectively integrate multiple copies of the transgene into AT-rich regions of host chromosomes, we successfully established six types of genetically modified 293 lines with different combinations of viral genes and/or OCT1. Through this methodology, we identified a stable 293 line (*i.e.*, RAPA) overexpressing both E1A and pTP driven by cytomegalovirus promoters,^92^ which can package and yield high titers of recombinant adenoviruses within 5 days under optimal transfection conditions.^21^ However, later studies revealed that the RAPA cell line tends to generate higher percentages of defective or non-infectious rAdV.

In this study, by stably overexpressing GAPDH in HEK293 (*i.e.*, 293G) and 293pTP (*i.e.*, 293GP) cells, we demonstrated that rAdV-induced ROS production and cell apoptosis were significantly diminished in 293G and 293GP cells. Transfection of 293G cells with pAd-G2Luc yielded much higher titers of Ad-G2Luc at as early as day 7 than that in HEK293 cells. Similarly, Ad-G2Luc was amplified more efficiently in 293G than in HEK293 cells. Furthermore, transfection of 293GP cells with pAd-G2Luc produced much higher titers of Ad-G2Luc at as early as day 5 than that of 293pTP cells, and 293GP cells amplified the Ad-G2Luc much more efficiently than 293pTP cells, indicating that exogenous GAPDH can further augment pTP-enhanced adenovirus production. Taken together, our results demonstrate that exogenous GAPDH can effectively suppress adenovirus-induced oxidative stress and ROS production, and subsequently accelerate adenovirus production. Thus, the engineered 293GP cells represent one of the fastest rAdV production systems that can significantly facilitate adenovirus-based gene transfer and gene therapy.

## Funding

The reported study was supported in part by research grants from the 10.13039/501100001809Natural Science Foundation of China (No. 82000744 to ZT, and 82102696 to J.F.), the Chongqing Bayu Young Scholar Award (China) (to J.F.), the 2019 Funding for Postdoctoral Research (10.13039/501100011786Chongqing Human Resources and Social Security Bureau of China) (No. 298 to J.F.), and the 10.13039/100000002National Institutes of Health (No. CA226303 to T.C.H., DE030480 to R.R.R.). W.W. was supported by the Medical Scientist Training Program of the National Institutes of Health (USA) (No. T32 GM007281). This project was also supported in part by The University of Chicago Cancer Center Support Grant (No. P30CA014599) and the 10.13039/100006108National Center for Advancing Translational Sciences of the 10.13039/100000002National Institutes of Health through grant number 2UL1TR002389-06 that funds the Institute for Translational Medicine (ITM). T.C.H. was supported by the Mabel Green Myers Research Endowment Fund and The University of Chicago Orthopaedics Alumni Fund. Funding sources were not involved in the study design; in the collection, analysis, and interpretation of data; in the writing of the report; and in the decision to submit the paper for publication.

## Author contributions

G.Z., W.T., J.M.F., Z.T., L.J., and T.C.H. conceived and designed the study. G.Z., P.Z., Y.W., H.Z., Y.Z., and J.Z. performed most of the experiments. G.Z. and T.C.H. collected and analyzed the data. W.Y., G.S., C.L., O.M., X.W., J.L., and Y.S. provided experimental resources and data validation. H.W., W.W., Y.B., L.L.S., and R.R.R. provided technical supervision and scientific advice. Z.T., J.F.,T.C.H., and R.R.R. secured funding support. G.Z., T.C.H., Z.T., J.F., R.R.R., H.H.L., Y.B., L.J., and W.T. drafted and revised the manuscript. All authors reviewed, edited, and approved the manuscript.

## Conflict of interests

The authors declared no conflict of interests.
