# Supplementary material for: GAPDH suppresses adenovirus-induced oxidative stress and enables a superfast production of recombinant adenovirus
Source: Genes Dis. 2024 May 31;11(6):101344. doi: 10.1016/j.gendis.2024.101344 (PMC11345542; doi:10.1016/j.gendis.2024.101344)
Supplement: Multimedia component 1 [file mmc1.pdf]

Glyceraldehyde 3-phosphate dehydrogenase (GAPDH) suppresses adenovirus-induced oxidative stress and enables a superfast production of recombinant adenovirus

Running Title: Exogenous GAPDH accelerates adenoviral production

Guozhi Zhao[1,2], Piao Zhao[1,2,3], Yonghui Wang[2,4], Hui Zhang[2,5], Yi Zhu[2,6], Jiamin Zhong[2,3], Wulin You[2,7], Guowei Shen[2,8], Changqi Luo[2,9], Ou Mei[2,10], Xingye Wu[1,2], Jingjing Li[2,11], Yi Shu[2,12], Hongwei Wang[13], William Wagstaff[2], Hue H. Luu[2], Yang Bi[2,12], Lewis L. Shi[2], Russell R. Reid[2,14], Tong-Chuan He[2,14], Li Jiang[1] *, Jiaming Fan[2,3] *, Wei Tang[1] *

1. Departments of Urology, Orthopedic Surgery, and Gastrointestinal Surgery, The First Affiliated Hospital of Chongqing Medical University, Chongqing, 400016, China
2. Molecular Oncology Laboratory, Department of Orthopaedic Surgery and Rehabilitation Medicine, The University of Chicago Medical Center, Chicago, IL 60637, USA
3. Ministry of Education Key Laboratory of Diagnostic Medicine, and Department of Clinical Biochemistry, School of Laboratory Medicine, Chongqing Medical University, Chongqing 400016, China
4. Department of Geriatrics, Xinhua Hospital, Shanghai Jiao-Tong University School of Medicine, Shanghai 200000, China
5. The Breast Cancer Center, Chongqing University Cancer Hospital, Chongqing 4000430, China
6. Department of Orthopaedic Surgery, Beijing Hospital, Chinese Academy of Medical Sciences & Peking Union Medical College, Beijing 100730, China
7. Department of Orthopaedic Surgery, Wuxi Hospital Affiliated to Nanjing University of Chinese Medicine, Wuxi 214071, China
8. Department of Orthopaedic Surgery, Benq Medical Center, The Affiliated Benq Hospital of Nanjing Medical University, Nanjing 210019, China
9. Department of Orthopaedic Surgery, Yibin Second People's Hospital, Affiliated with West China School of Medicine, Yibin 644000, China
10. Department of Orthopedics, Jiangxi Hospital of Traditional Chinese Medicine, Jiangxi University of Traditional Chinese Medicine, Nanchang 330006, China
11. Department of Oncology, The Affiliated Hospital of Weifang Medical University, Weifang 261053, China
12. Stem Cell Biology and Therapy Laboratory of the Pediatric Research Institute, the National Clinical Research Center for Child Health and Disorders, and Ministry of Education Key Laboratory of Child Development and Disorders, the Children's Hospital of Chongqing Medical University, Chongqing 400016, China
13. Division of Research and Development, Decoding Therapeutics, Inc., Mt Prospect, IL 60056, USA
14. Laboratory of Craniofacial Biology and Development, Department of Surgery Section of Plastic Surgery, The University of Chicago Medical Center, Chicago, IL 60637, USA

* Corresponding authors.

CORRESPONDENCES

Wei Tang, MD
Department of Urology
The First Affiliated Hospital
Chongqing Medical University
Chongqing, 400016, China
Email: wei-tang@boneandcancer.org

Jiaming Fan, MD, PhD
Ministry of Education Key Laboratory of Diagnostic Medicine
Department of Clinical Biochemistry
School of Laboratory Medicine
Chongqing Medical University
Chongqing, 400016, China
Tel. 011-86-23-6848 5240
Email: fanjiaming1988@cqmu.edu.cn

Li Jiang, MD, PhD
Department of Urology
The First Affiliated Hospital
Chongqing Medical University
Chongqing, 400016, China
Email: jiangli0529@163.com

SUPPORTING MATERIALS

Table S1. Oligonucleotides Used in the Study

Gene	Nucleotide Sequence	Accession No	Use
GAPDH	ggaGGATCCaccaccATGGGGAAGGTGAAGGTCGGAGTC<br>acgACGCGTTTACTCCTTGGAGGCCATGTGGGC<br>GGGAAACTGTGGCGTGATGGCCGC	NM_002046	cloning
	GACCCCTTCATTGACCTCAACT<br>TGCTGATGATCTTGAGGCTGTT<br>ACAGCCTCAAGATCATCAGCAA<br>TGCTTCACCACCTTCTTGATGT		semi-qPCR
	GTGGTGGACCTGACCTGC<br>TGTAGCCCAGGATGCCCT<br>AGGGCATCCTGGGCTACA<br>CCTTGGAGGCCATGTGGG		TqPCR
TBP	AGCAGTCAACGTCCCAGC<br>TGGGAGTCATGGGGGAGG	NM_003194.5	
HPRT1	CTCAGGCGAACCTCTCGG<br>TAATCACGACGCCAGGGC	NM_000194.3	

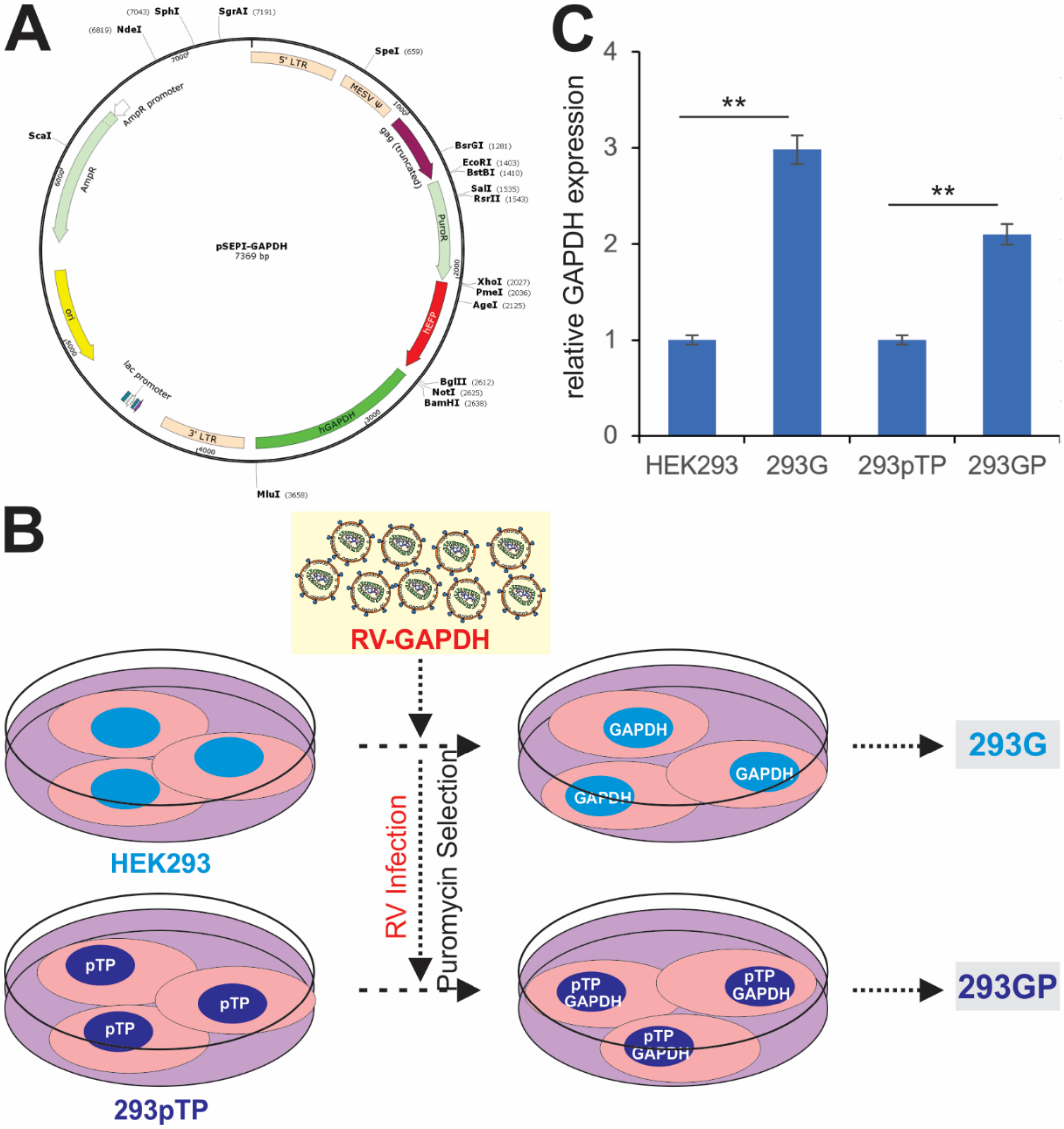

Figure S1. **Establishment of stable GAPDH expression lines for adenovirus packaging and amplification**. (**A**) Schematic representation of the retroviral vector pSEPI-GAPDH, in which GAPDH expression is driven by the strong and constitutive promoter hEFH, with puromycin resistance as a selection marker. (**B**) Flowchart for the generation of 293G and 293GP lines. Parental HEK293 cell and previously established pTP expression 293 derivatives 293pTP were infected with packaged retroviral particle RV-GAPDH, followed by puromycin selection. The resultant stable clones are designated as 293G and 293GP lines, respectively. (**C**) Verification of exogenous GAPDH expression. Total RNA was isolated from subconfluent HEK293, 293G, 293pTP and 293GP cells, and subjected to TqPCR analysis using human GAPDH specific primer pairs. *TBP* and *HPRT1* were used as internal references. “**” $p < 0.01$, compared with that of the HEK293 and 293pTP group, respectively.

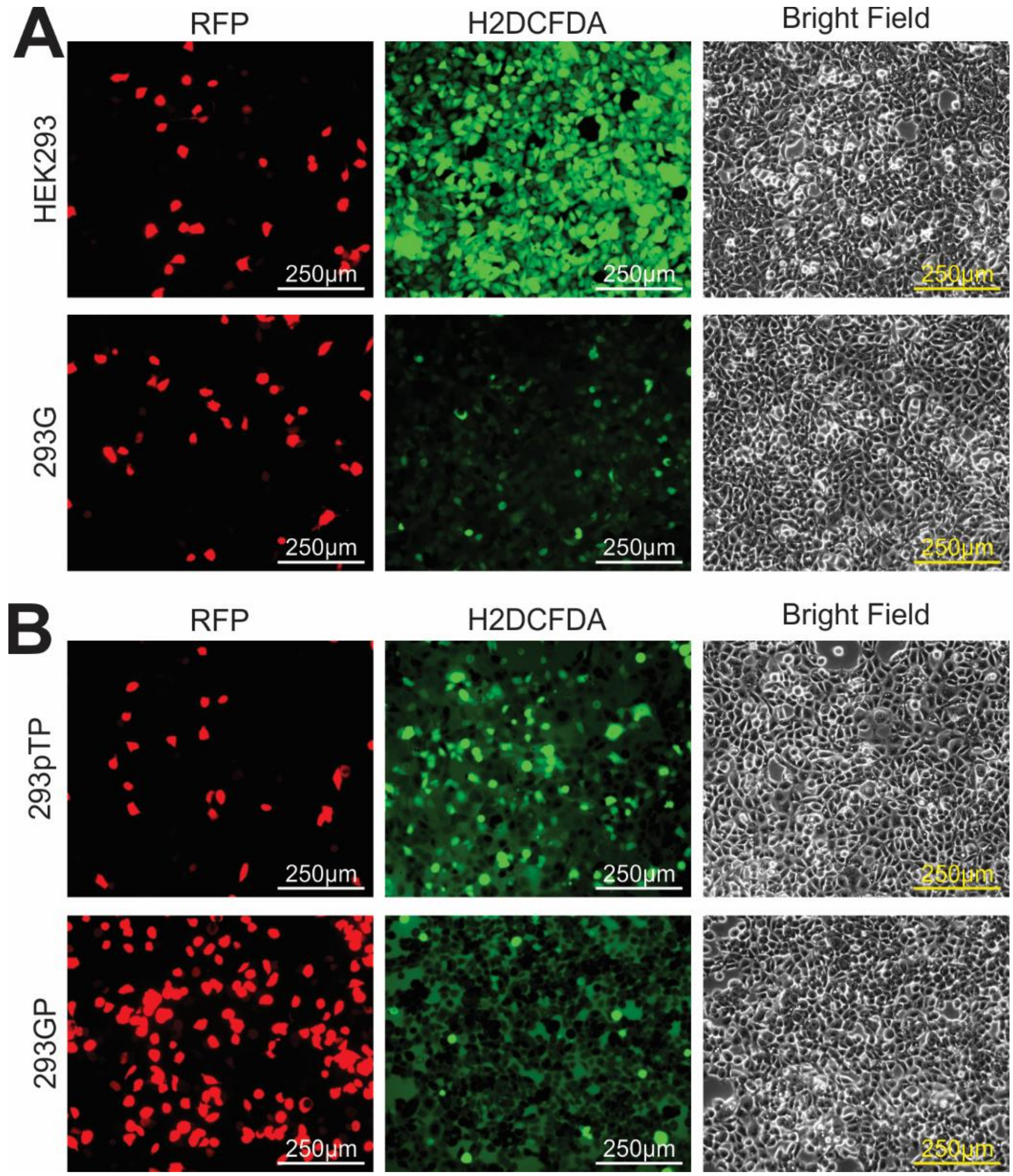

Figure S2. **Exogenously expressed GAPDH effectively curtails adenovirus-induced production of reactive oxidative species (ROS) in the adenovirus packaging cells.** Subconfluent HEK293 and 293G cells (**A**) or 293pTP and 293GP cells (**B**) were infected with the same titer of Ad-RFP for 36h, ROS biosensor H2DCFDA (5 μM) was added to the infected cells for 30 min, followed by RFP and GFP imaging at a lower magnification, compared with that of **Figure 1**. Bright field images were also recorded under transmissive light. Representative images are shown.

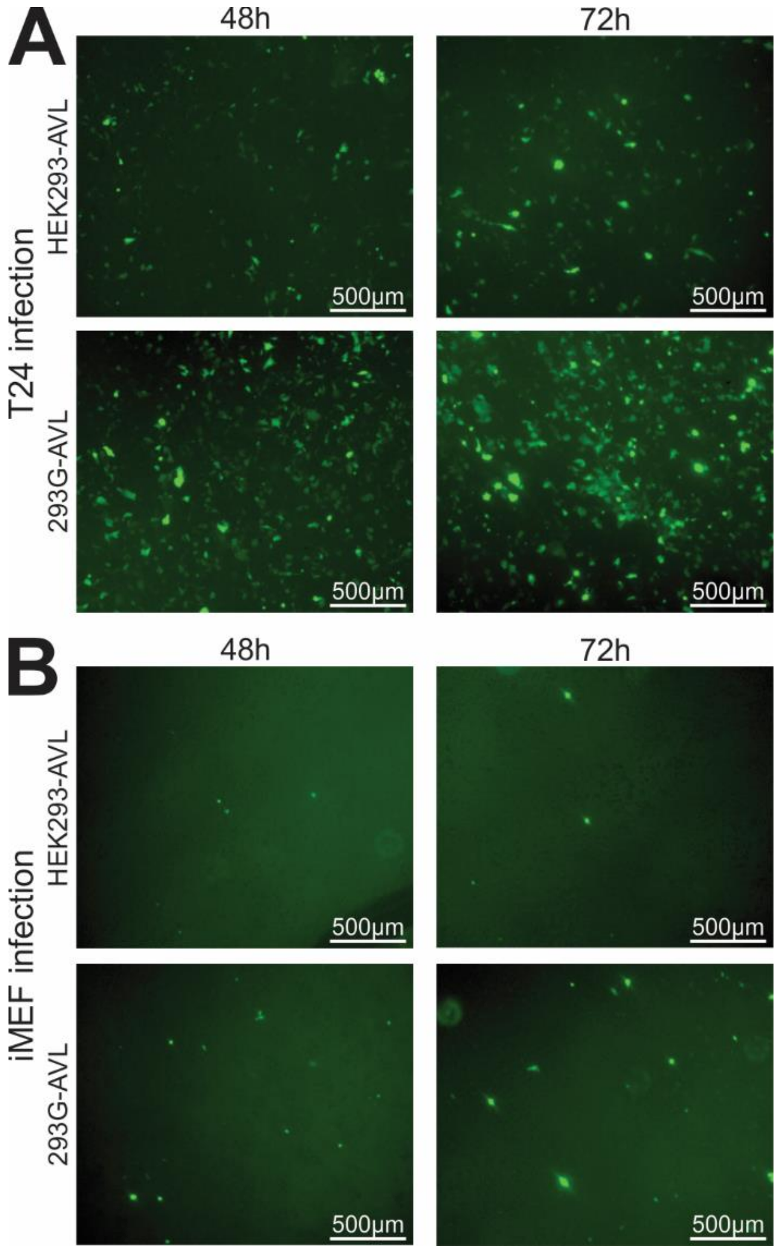

Figure S3. **Exogenous GAPDH expression in HEK293 cells enhances the amplification and production of recombinant adenovirus**. Equal % of the amplification viral lysate (AVL) prepared from HEK293 (i.e., HEK293-AVL) and 293G (i.e., 293G-AVL) (as shown in **Figure 4**) was used to infect subconfluent T24 cells (**A**) and iMEFs (**B**), while GFP signal was weak in USCs (data not shown). Representative images are shown.

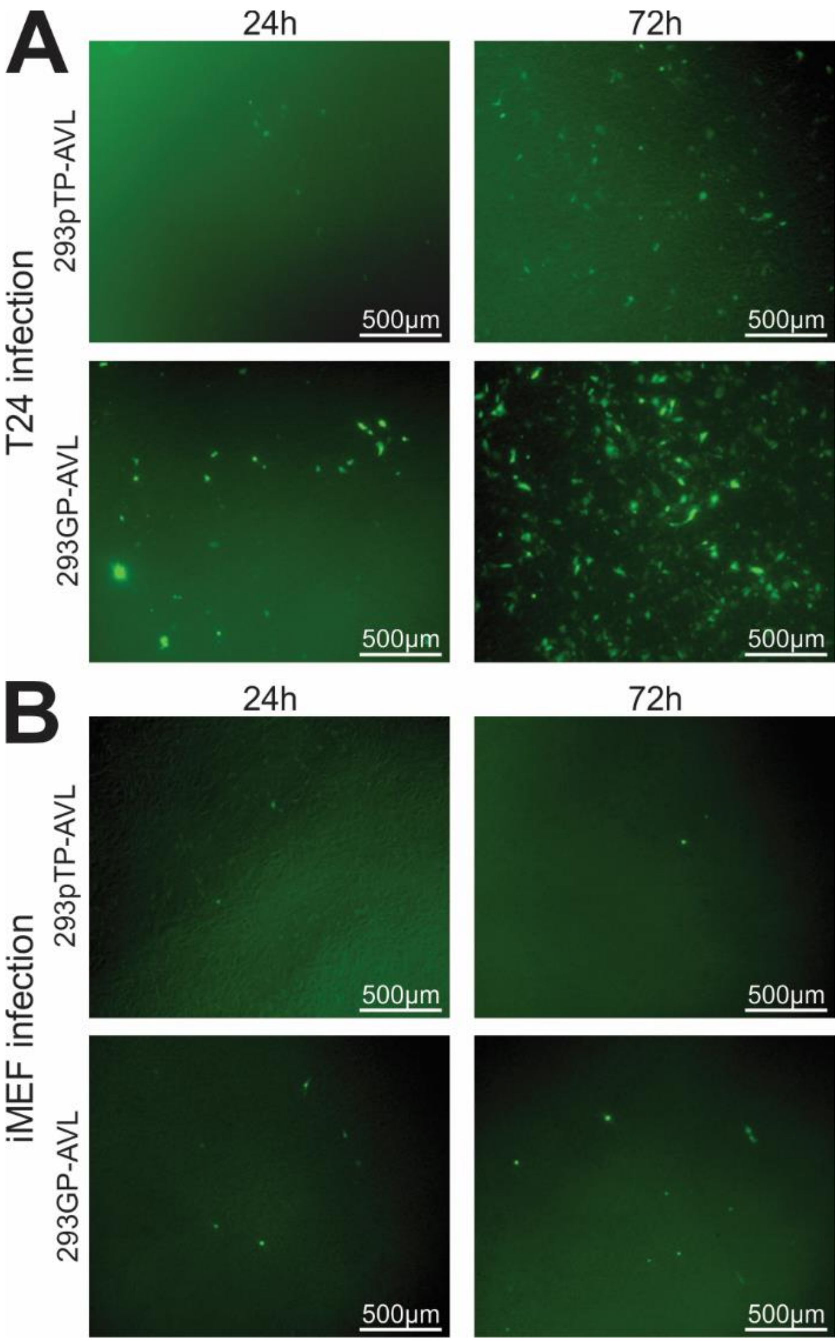

Figure S4. **Exogenous GAPDH expression further augments pTP-enhanced amplification and production of recombinant adenovirus**. Equal % of the amplification viral lysate (AVL) prepared from 293pTP (i.e., 293pTP-AVL) and 293GP (i.e., 293GP-AVL) (as shown in **Figure 6**) was used to infect subconfluent T24 cells (**A**) and iMEFs (**B**), while GFP signal was weak in USCs (data not shown). Representative images are shown.
